# Efficacy and safety of oral ibrexafungerp for the treatment of acute vulvovaginal candidiasis: a global phase 3, randomised, placebo‐controlled superiority study (VANISH 306)

**DOI:** 10.1111/1471-0528.16972

**Published:** 2021-11-08

**Authors:** R Sobel, P Nyirjesy, MA Ghannoum, DA Delchev, NE Azie, D Angulo, IA Harriott, K Borroto‐Esoda, JD Sobel

**Affiliations:** ^1^ Department of Obstetrics and Gynecology Jefferson Vulvovaginal Health Center Sidney Kimmel Medical College Thomas Jefferson University Philadelphia PA USA; ^2^ Department of Dermatology Center for Medical Mycology Case Western Reserve University and University Hospitals Cleveland Medical Center Cleveland OH USA; ^3^ Department of Gynaecology MHAT Dr. Bratan Shukerov AD Smolyan Bulgaria; ^4^ Departments of Clinical Development and Medical Affairs SCYNEXIS, Inc. Jersey City NJ USA; ^5^ Department of Clinical Research SCYNEXIS, Inc. Jersey City NJ USA; ^6^ Department of Medical Affairs SCYNEXIS, Inc. Jersey City NJ USA; ^7^ KBE Consulting Raleigh NC USA; ^8^ Infectious Diseases Department of Internal Medicine Wayne State University Detroit MI USA

**Keywords:** Antifungal, ibrexafungerp, vulvovaginal candidiasis

## Abstract

**Objective:**

To evaluate the efficacy and safety of ibrexafungerp versus placebo for acute vulvovaginal candidiasis (VVC) treatment.

**Design:**

Global phase 3, randomised, placebo‐controlled superiority study.

**Setting:**

Study sites in the USA (*n* = 19) and Bulgaria (*n* = 18).

**Population:**

Female patients aged ≥12 years with acute VVC and a vulvovaginal signs and symptoms (VSS) score ≥4 at baseline.

**Methods:**

Patients were randomly assigned 2:1 to ibrexafungerp (300 mg twice for 1 day) or placebo.

**Main outcome measures:**

The primary endpoint was the percentage of patients with a clinical cure (VSS = 0) at the test‐of‐cure visit (day 11 ± 3). Secondary endpoints included percentages of patients with mycological eradication, clinical cure and mycological eradication (overall success), clinical improvement (VSS ≤1) at test‐of‐cure visit, and complete resolution of symptoms at follow‐up visit (day 25 ± 4).

**Results:**

At the test‐of‐cure visit, patients receiving ibrexafungerp had significantly higher rates of clinical cure (63.3% [119/188] versus 44.0% [37/84]; *P* = 0.007), mycological eradication (58.5% [110/188] versus 29.8% [25/84]; *P* < 0.001), overall success (46.1% [82/188] versus 28.4% [23/84]; *P* = 0.022) and clinical improvement (72.3% [136/188] versus 54.8% [46/84]; *P* = 0.01) versus those receiving placebo. Symptom resolution was sustained and further increased with ibrexafungerp (73.9%) versus placebo (52.4%) at follow‐up (*P* = 0.001). Ibrexafungerp was generally well tolerated. Adverse events were primarily gastrointestinal and were mild to moderate in severity.

**Conclusions:**

Ibrexafungerp demonstrated statistical superiority over placebo for the primary and secondary endpoints. Ibrexafungerp is a promising novel, well‐tolerated and effective oral 1‐day treatment for acute VVC.

**Tweetable abstract:**

Ibrexafungerp is statistically superior to placebo for the treatment of vulvovaginal candidiasis.

## Tweetable abstract

Ibrexafungerp is statistically superior to placebo for the treatment of vulvovaginal candidiasis.

**Linked article** This article is commented on by KSJ Olah, p. 421–422 in this issue. To view this mini commentary visit https://doi.org/10.1111/1471-0528.17000.

## Introduction

Vulvovaginal candidiasis (VVC) is the second most common cause of vaginitis worldwide, affecting women of all races/ethnicities and socio‐economic status, with ~90% of VVC caused by *Candida albicans*.^1^, ^2^ Despite the commonality of VVC, its epidemiology is variable, with recently reported incidence rates ranging from 12.1 to 57.3% across various countries.^3^, ^4^ Historically, vulvovaginal candidiasis treatment has been largely limited to the azole class of fungistatic agents.^5^ Prescription and over‐the‐counter antifungals are available in oral and intravaginal formulations, all of which are generally effective for patients with uncomplicated disease.^5^, ^6^, ^7^ However, treatment options are limited for women with VVC caused by azole‐resistant *Candida* species and for those who are intolerant to or have a contraindication for azoles. New treatment approaches are needed to provide broad‐spectrum fungicidal activity with favourable safety and limited drug‐drug interactions.

Ibrexafungerp is a first‐in‐class, orally active, semisynthetic, triterpenoid derivative that blocks the synthesis of the fungal cell wall polymer β‐(1,3)‐d‐glucan.^8^, ^9^, ^10^ Ibrexafungerp has *in vitro* fungicidal activity against different *Candida* species strains, including those that are echinocandin‐ and azole‐resistant.^11^ *In vitro* studies^10^, ^12^ with ibrexafungerp have shown potentiation of antifungal activity against *C. albicans* at vaginal pH levels (4.5) that are consistent with VVC infections, as compared with a standard laboratory pH level of 7.0, and have shown a high potential for ibrexafungerp to accumulate in vaginal tissues and fluid. In the phase 2 DOVE study,^13^ treatment with ibrexafungerp (300 mg twice daily [BID] on 1 day) was selected as the dose to be evaluated in phase 3 studies based on an observed increase in gastrointestinal‐related adverse events without a corresponding improvement in efficacy as total milligram dosing increased.

Pivotal studies of ibrexafungerp have been designed in accordance with U.S. Food and Drug Administration guidance^14^ for industry that was issued in 2019 to support the development of drugs for treating VVC. Here we report results from the global phase 3 VANISH 306 study (www.clinicaltrials.gov NCT03987620) that evaluated the efficacy and safety of oral ibrexafungerp for the treatment of VVC. Based on current guidance, the efficacy assessments in this study differ from those previously reported in VVC and include stricter criteria for a clinical cure (absence of all vulvovaginal signs and symptoms [VSS] or VSS = 0) at follow‐up (FU) visits between days 7–14 and 21–30.^14^, ^15^, ^16^

## Methods

### Participants

VANISH 306 is a global, multicentre, randomised, double‐blind, placebo‐controlled study that evaluated the efficacy and safety of oral ibrexafungerp versus placebo in postmenarchal females aged ≥12 years with moderate to severe VVC, defined as a VSS score ≥4 at baseline, with at least two signs or symptoms having a score of ≥2. Patients were not involved in the development of this research. Eligible participants were required to have normal vaginal pH levels (≤4.5) and a positive result on microscopic examination with 10% potassium hydroxide of a vaginal sample collected at screening. Participants were excluded if they were pregnant, lactating or likely to become pregnant; had a vaginal condition other than acute VVC that may have interfered with diagnosis or evaluation of response to therapy, including mixed infections; had received systemic and/or topical antifungal treatment within 28 days of baseline; had active menstruation at baseline; had uncontrolled diabetes mellitus; had a history of or active cervical or vaginal cancer; had a known HIV infection; or had an illness or were receiving therapy that induced an immune deficiency.

This study was conducted in accordance with the ethical principles of the Declaration of Helsinki, the International Council for Harmonisation of Technical Requirements for Pharmaceuticals for Human Use tripartite guideline ‘E6: Good Clinical Practice’, the U.S. Code of Federal Regulations, applicable European regulations, and/or other national and local ethical and legal requirements, as applicable. Each study site obtained institutional review board/ethics committee approval before study initiation, and each patient provided written consent for study participation.

### Study design

Patients were randomly assigned in a 2:1 ratio to receive ibrexafungerp 300 mg as two 150‐mg tablets or matching placebo tablets (two tablets administered BID for 1 day). At randomisation, patients were stratified based on the presence of diabetes mellitus. Randomisation was performed using an interactive voice‐ or web‐based response system, which assigned a unique randomisation number for each patient corresponding to a study treatment (block size = 6). Efficacy analyses were also reported by country. All patients and site and sponsor personnel were blinded to treatment assignment, except for a sponsor representative who was involved with drug distribution logistics. To maintain blinding, the active and placebo dose forms had similar appearance.

Patients used a diary to rate their vulvovaginal symptoms and record study drug dosing details, adverse events (AEs), and concomitant medication use daily from day 1 through the test‐of‐cure (TOC) visit. All patients continued in the study unless they withdrew consent, were lost to FU, or experienced an AE that warranted discontinuation or the investigator believed it was in their best interest to withdraw from the study.

### Assessments

This study included baseline, TOC (day 11 ± 3), and FU (day 25 ± 4) visits. Clinical evaluations included patient diary ratings of VVC symptoms daily from day 1 to TOC visit and at the FU visit, as well as investigator VVC signs ratings based on physical examinations performed at baseline and TOC visits and again at FU if the patients were symptomatic. Patients rated symptoms of burning, itching and irritation, and investigators rated the signs of oedema, erythema and excoriation/fissures on a scale from 0 = absent to 3 = severe; total composite scores range from 0 to 18.

Mycological testing included direct microscopic examination with 10% potassium hydroxide and fungal cultures. Potassium hydroxide tests were performed at a local laboratory at screening (to determine patient eligibility) and at the TOC visit and at the FU visit if symptoms persisted or recurred. Vulvovaginal samples were obtained at screening for local vaginal pH determination, and these samples were evaluated for bacterial vaginosis and *Trichomonas vaginalis*. If herpes virus, *Neisseria gonorrhoeae* or *Chlamydia trachomatis* infection was suspected, samples were evaluated at a local or central laboratory. Vaginal samples for fungal cultures were collected at screening, at TOC visits and before initiation of rescue antifungal medication, and if patients were symptomatic at the FU visit. Fungal cultures were assessed by qualified central laboratories per Clinical & Laboratory Standards Institute (CLSI) M27‐A3 guidelines and European Committee on Antimicrobial Susceptibility Testing (EUCAST) E.DEF 7.3.1 methods.

If a patient had persistent, worsening, or recurrent symptoms (e.g. ≥3 symptoms), then rescue antifungal therapy was allowed. If rescue antifungal therapy was administered, the patient was considered an early termination due to lack of efficacy.

Safety assessments included continuous AE monitoring, physical examinations, vital sign measurements, laboratory testing, and review of prior and concomitant medications.

### Outcomes

The primary study objective was to evaluate the clinical efficacy of oral ibrexafungerp versus placebo in patients with acute VVC, with efficacy based on the percentage of patients who reached clinical cure (VSS = 0) at the TOC visit. Secondary endpoints included the percentage of patients with mycological eradication at the TOC visit, the percentage of patients with both clinical cure and mycological eradication (overall success) at the TOC visit, the percentage of patients with complete resolution of symptoms at the FU visit, the percentage of patients with clinical improvement (VSS ≤1) at the TOC visit, and safety and tolerability. Adverse events were coded using Medical Dictionary for Regulatory Activities (version 21.1). Efficacy endpoint definitions are provided in Table 1.

**Table 1:** Efficacy endpoint definitions

Endpoint	Definition
Clinical cure	Complete resolution of signs and symptoms of vulvovaginal infection without need for further antifungal treatment and topical vaginal drug therapy for the treatment of vulvovaginal irritation/pruritis before or at the TOC visit. VSS = 0 at TOC visit
Complete resolution of symptoms at FU visit	Complete resolution of symptoms in patients at FU visit regardless of clinical cure at TOC visit without need for further antifungal treatment or topical vaginal drug therapy for the treatment of vulvovaginal irritation/pruritus before or at the FU visit. Symptom score = 0 at FU visit
Clinical improvement	Partial or complete resolution of signs and symptoms with total composite score ≤1 at TOC visit without need for further antifungal treatment and topical drug therapy for the treatment of vulvovaginal irritation/pruritis before or at the TOC visit. VSS score ≤1 at TOC visit
Mycological eradication	Negative culture for *Candida* species without the need for further antifungal treatment at TOC visit
Overall success	Clinical cure and mycological eradication at TOC visit

FU, follow‐up; TOC, test‐of‐cure; VSS, vulvovaginal signs and symptoms.

Considering that positive clinical outcome has previously been defined as VSS ≤2 in other studies,^15^, ^17^ we conducted a *post hoc* analysis to evaluate the percentage of patients with VSS ≤2 at the TOC visit.

### Statistical analysis

All analyses were conducted using SAS software (version 9.4; SAS Institute Inc., Cary, NC, USA). A sample size of 282 patients with a mycological culture‐confirmed infection at baseline was calculated to provide 90% power to detect an ibrexafungerp–placebo difference based on a Pearson chi‐square test with a type 1 error rate of 5% with an assumed 50% and 30% clinical cure rate for ibrexafungerp and placebo, respectively, administered in a 2:1 ratio. The percentage of patients who would have a negative culture was estimated at approximately 20% and an additional 72 patients were added for a total of 354 patients (ibrexafungerp, *n* = 236; placebo, *n* = 118). The protocol was amended to allow up to 470 patients to be enrolled, to achieve 282 evaluable patients secondary to a higher than anticipated rate of ‘no growth’ baseline samples.

Disposition was recorded for all randomised patients who received ≥1 dose of study drug (intention‐to‐treat population). Efficacy analyses were performed using the modified intention‐to‐treat (mITT) population (i.e. all randomised patients who had a positive culture for *Candida* species at baseline and received ≥1 dose of study drug). Safety was evaluated for all patients who received ≥1 dose of study drug and had ≥1 postbaseline evaluation.

Patients were considered to be nonresponders if they did not meet the clinical response criteria (VSS = 0) for categorical responses or were missing categorical response data at specific visits. Treatment differences between ibrexafungerp and placebo were compared using the Cochran–Mantel–Haenszel test adjusted for country and diagnosis of diabetes mellitus.

For each treatment group and treatment comparison versus placebo, least squares mean, SE, 95% CI and *P*‐value were presented. Continuous variables were summarised descriptively, and categorical variables were summarised using patient counts and percentages. All statistical tests were 2‐sided and interpreted at a 5% level of significance. Subgroup analyses were performed based on country (USA and Bulgaria).

### Role of the funding source

SCYNEXIS, Inc. (Jersey City, NJ, USA) supported this study and was responsible for working with the authors in the development of the protocols; in the collection, analysis and interpretation of study data; in the writing of the clinical study report; and in the decision to submit the article for publication.

## Results

**Figure 1:**
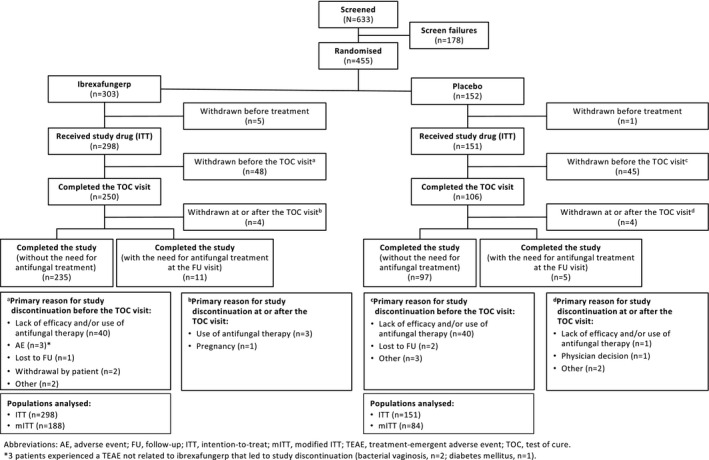
Patient disposition.

Patients were enrolled between 7 June 2019 and 7 February 2020, at 18 study sites in Bulgaria and 19 study sites in the USA. Patient disposition is summarised in Figure 1. A total of 633 patients were assessed for eligibility, of whom 449 were included in the intention‐to‐treat population and randomly assigned to treatment and received ≥1 dose of ibrexafungerp (*n* = 298) or placebo (*n* = 151). Of these patients, 272 were included in the mITT population (ibrexafungerp, *n* = 188; placebo, *n* = 84).

Demographic characteristics, including the severity of acute VVC at baseline, were similar between treatment groups (Table 2); 4.3% and 6.0% of patients receiving ibrexafungerp and placebo, respectively, had diabetes mellitus. Median VSS score at baseline was 10.0 (range, 4–18) with ibrexafungerp and 10.0 (range, 5–18) with placebo.

**Table 2:** Baseline demographic and disease characteristics (mITT population)

	Ibrexafungerp (*n* = 188)	Placebo (*n* = 84)
Age, y		
Mean ± SD	33.7 ± 10.3	33.5 ± 10.4
Median (min, max)	32.0 (18, 65)	32.0 (18, 65)
Race, *n* (%)		
White	153 (81.4)	69 (82.1)
Black or African American	34 (18.1)	15 (17.9)
American Indian or Alaska Native	1 (0.5)	0
Ethnicity, *n* (%)		
Hispanic or Latino	21 (11.2)	6 (7.1)
Not Hispanic or Latino	167 (88.8)	78 (92.9)
Country, *n* (%)		
Bulgaria	122 (64.9)	48 (57.1)
USA	66 (35.1)	36 (42.9)
Body mass index (kg/m^2^)*		
≤35	167 (88.8)	69 (82.1)
>35	21 (11.2)	15 (17.9)
Diabetes mellitus		
Yes	8 (4.3)	5 (6.0)
No	180 (95.7)	79 (94.0)
*Candida* species		
*Candida albicans*	165 (87.8)	76 (90.5)
*Candida glabrata*	20 (10.6)	8 (9.5)
*Candida tropicalis*	3 (1.6)	3 (3.6)
*Candida kefyr*	3 (1.6)	1 (1.2)
*Candida parapsilosis*	3 (1.6)	0
*Candida dubliniensis*	0	1 (1.2)
*Candida krusei*	2 (1.1)	0
*Candida inconspicua*	1 (0.5)	0
*Candida lusitaniae*	1 (0.5)	0
*Candida norvegensis*	1 (0.5)	0

max, maximum; min, minimum; mITT, modified intention‐to‐treat; VSS, vulvovaginal signs and symptoms.

* Baseline body mass index is calculated as baseline weight (kg)/baseline height (m^2^).

All patients in the mITT population had a positive culture for ≥1 *Candida* species at baseline, with most testing positive for *C. albicans* (ibrexafungerp, 87.8%; placebo, 90.5%). Baseline susceptibility testing showed no notable differences between treatment groups. No fluconazole‐resistant isolates of *C. albicans* were identified at baseline using CLSI and EUCAST methods. Evaluation of isolates obtained at TOC visit showed no change in susceptibility following ibrexafungerp exposure.

**Figure 2:**
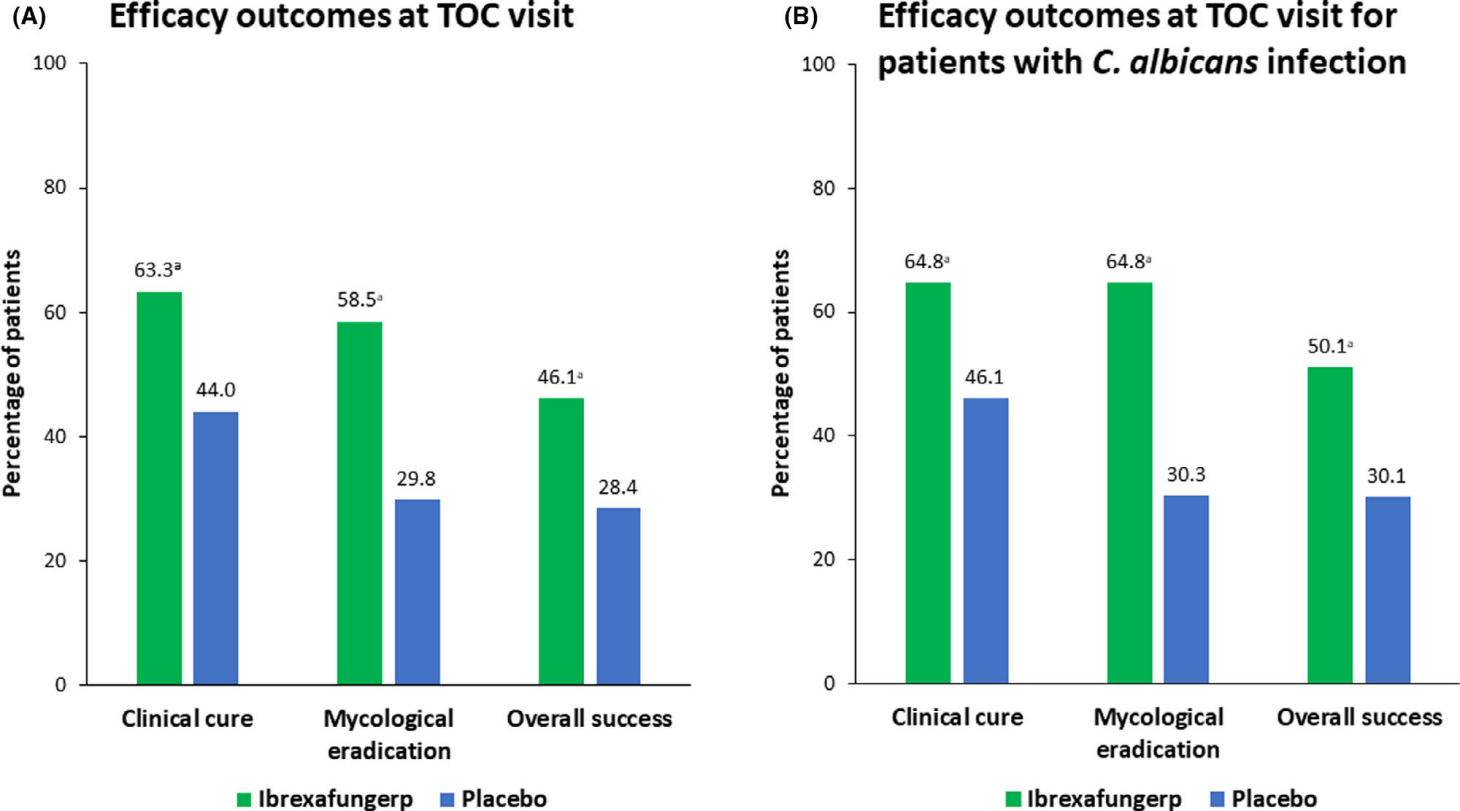
Select efficacy endpoints. (A) Efficacy outcomes at TOC visit (day 10): clinical cure (ibrexafungerp, 119 of 188 patients, versus placebo, 37 of 84 patients; *P* = 0.007), mycological eradication (ibrexafungerp, 110 of 188 patients, versus placebo, 25 of 84 patients; *P* < 0.001), and overall success (ibrexafungerp, 82 of 178 patients, versus placebo, 23 of 81 patients; *P* = 0.022). (B) Efficacy outcomes at TOC visit (day 10) for patients with *Candida albicans* infection: clinical cure (ibrexafungerp, 107 of 165 patients, versus placebo, 35 of 76 patients; *P* = 0.013), mycological eradication (ibrexafungerp, 107 of 165 patients, versus placebo, 23 of 76 patients; *P* < 0.001), and overall success (ibrexafungerp, 80 of 157 patients, versus placebo, 22 of 73 patients; *P* = 0.009). ^a^Significant difference for comparison between ibrexafungerp and placebo. TOC, test‐of‐cure.

Ibrexafungerp demonstrated statistical superiority over placebo in the primary endpoint and all key secondary endpoints. The clinical cure rate at TOC visit was significantly higher in patients receiving ibrexafungerp (63.3% [119 of 188]) than in those receiving placebo (44.0% [37 of 84]; relative risk [RR] 1.38; 95% CI 1.073–1.783; *P* = 0.007) (Figure 2A). The clinical cure rate at the TOC visit for patients with *C. albicans* infection was also significantly higher in patients receiving ibrexafungerp than in those receiving placebo (RR 1.35; 95% CI 1.046–1.744; *P* = 0.013) (Figure 2B). A higher clinical cure rate at the TOC visit was observed with ibrexafungerp compared with placebo in both the US subgroup (54.5% [36 of 66] of patients versus 27.8% [10 of 36] of patients, respectively) and the Bulgarian subgroup (68.0% [83 of 122] of patients versus 56.3% [27 of 48] of patients, respectively). Although clinical cure was defined as VSS = 0 in our study, the percentage of patients with clinical improvement (VSS ≤1) at TOC visit was also significantly higher with ibrexafungerp (72.3% [136 of 188]) than with placebo (54.8% [46 of 84]; RR 1.28; 95% CI 1.043–1.570; *P* = 0.010). In a *post hoc* analysis, in patients receiving ibrexafungerp, the clinical cure rate using VSS ≤2 was 76.1% (143 of 188).

The percentage of patients with complete symptom resolution at the FU visit—without having received rescue antifungal treatment and regardless of having achieved a clinical cure at the TOC visit—was also significantly higher with ibrexafungerp (73.9% [139 of 188]) than with placebo (52.4% [44 of 84]; *P* = 0.001). Significant results were also seen in patients with *C. albicans* at baseline, with complete symptom resolution occurring in 77.0% (127 of 165) of patients receiving ibrexafungerp versus 52.6% (40 of 76) of patients receiving placebo (*P* < 0.001).

The mycological eradication rate (negative culture for *Candida* species) at TOC visit was significantly higher with ibrexafungerp than with placebo (RR 1.85; 95% CI 1.329–2.583; *P* < 0.001) (Figure 2A). Patients receiving ibrexafungerp who tested positive for *C. albicans* at baseline also had a significantly higher rate of mycological eradication compared with the placebo group at TOC visit (RR 2.01; 95% CI 1.439–2.814; *P* < 0.001) (Figure 2B). Mycological samples were not required for patients without symptoms at FU and were only collected in 7.4% of patients receiving ibrexafungerp and 17.9% of patients receiving placebo.

The overall therapeutic success rate was significantly higher with ibrexafungerp than with placebo (RR 1.48; 95% CI 1.038–2.113; *P* = 0.022) (Figure 2A). Patients receiving ibrexafungerp who tested positive for *C. albicans* at baseline had a significantly higher rate of overall success than the placebo group did (RR 1.56; 95% CI 1.091–2.219; *P* = 0.009) (Figure 2B).

Overall, ibrexafungerp was well tolerated, with 44 of 298 patients (14.8%) reporting a treatment‐related treatment‐emergent adverse event (TEAE) compared with six of 151 patients (4.0%) in the placebo group. The most frequently reported treatment‐related TEAEs were gastrointestinal‐related and mild to moderate in intensity. Treatment‐related TEAEs occurring in ≥2% of patients receiving ibrexafungerp were nausea (6.4% mild, 0.3% moderate, 0.3% severe) and diarrhoea (5.7% mild, 1.0% moderate). No treatment‐related TEAEs occurred in ≥2% of the placebo group. A lower percentage of patients experienced a TEAE in the Bulgarian subgroup (ibrexafungerp, [19.2% [37 of 193]; placebo, 21.2% [18 of 85]) than in the US subgroup (ibrexafungerp, 59.0% [62 of 105]; placebo, 39.4% [26 of 66]).

Two patients receiving ibrexafungerp reported three treatment‐related TEAEs leading to dose interruption (abdominal pain, *n* = 2; vomiting, *n* = 1). No patients in the placebo group discontinued treatment or the study. No treatment‐related serious AEs or deaths were reported. One pregnancy was reported in the ibrexafungerp group on study day 12. The pregnancy was electively terminated approximately 2 weeks later.

## Discussion

### Main findings

The VANISH 306 study extends the results of the US‐based VANISH 303 study (NCT03734991).^18^ Similar to VANISH 303, results of this study showed that ibrexafungerp was well tolerated and superior to placebo in clinical cure rates at the TOC visit. In line with the reported epidemiology for this condition,^1^, ^2^ most patients in this study had VVC caused by *C. albicans* (87.8% and 90.5% in the ibrexafungerp and placebo groups, respectively). As expected, the efficacy conclusions in patients with *C. albicans* infections are consistent with the overall efficacy conclusions. Clinical improvement (VSS ≤1) at the TOC visit was also significantly higher with ibrexafungerp and, regardless of clinical cure at the TOC visit, complete resolution of symptoms at the FU visit was significantly higher than with placebo. The efficacy of ibrexafungerp was evident at the TOC visit, with significantly higher mycological eradication and overall success rates compared with those with placebo. Ibrexafungerp was well tolerated, with most treatment‐related TEAEs being gastrointestinal in nature and mild to moderate in severity.

### Strengths and limitations

Although our study is limited by the use of a placebo arm, it provides relevant information for research‐planning purposes, regarding placebo response rates in a large study in VVC, which was lacking in the literature. Our study is also limited by a lack of racial/ethnic diversity and low numbers of patients with a body mass index >35. Although females ≥12 years of age were eligible for inclusion in this study, no one <18 years of age was enrolled. Evaluation of ibrexafungerp in patients <18 years of age will need to be evaluated in future studies. Finally, the study included only a small number of patients with non‐*albicans* infections (Table 2), though proportional to epidemiologically reported rates, thereby limiting efficacy determinations in these non‐*albicans* species.

### Interpretation

This is the third clinical study to evaluate the efficacy of ibrexafungerp in acute VVC. The clinical cure (VSS = 0) rates with ibrexafungerp versus placebo in this study (63.3% and 44.0%, respectively) were notably higher than those observed in the VANISH 303 study^18^ (50.5% and 28.6%, respectively) and were higher than with ibrexafungerp and fluconazole in the phase 2 DOVE study^13^ (51.9% and 58.3%, respectively). The higher placebo clinical cure rate in this study is consistent with a 45% placebo response rate reported in a small study of itraconazole.^19^ However, the current study assumed a 30% placebo clinical cure rate. Differences in response rates in this study compared with those in VANISH 303 were driven by differences in responses between US and Bulgarian participants. In the current study, clinical cure rates in the ibrexafungerp and placebo groups, respectively, were 54.5% and 27.8% among US participants, similar to those reported in the US‐based VANISH 303 study. In Bulgarian participants, clinical cure rates were higher for both ibrexafungerp and placebo (68.0% and 56.3%, respectively). It is unclear why higher clinical cure rates were reported in Bulgaria participants for both ibrexafungerp and placebo, but regional differences in global clinical trials are not uncommon. In general, the analysis of outcome in each region supported the overall conclusions of the study.

In accordance with pharmaceutical industry guidance issued by the U.S. Food and Drug Administration in 2019, clinical cure (i.e. complete resolution of signs and symptoms of vulvovaginal infection without need for further antifungal treatment or topical vaginal drug therapy for the treatment of vulvovaginal irritation/pruritus before or at TOC) was selected as the primary efficacy endpoint of this study. Mycological eradication was not a primary endpoint, as *Candida* can normally reside in a healthy host.^20^ Historical comparisons with current therapies for VVC are difficult because of differences in dosing regimens and study methodology, including the definition of clinical cure—VSS = 0 in this study versus VSS ≤ 2 in many other azole studies. Clinical cure (VSS = 0) rates of 47.4% and 57.9% on days 7 and 14 have been reported in patients receiving single‐dose fluconazole^21^ versus 63.3% with single‐day ibrexafungerp in our study. With single‐dose fluconazole,^17^ a clinical cure (VSS ≤2) rate of 80.9% on day 14 was reported versus 76.1% with single‐dose ibrexafungerp in our study. Additionally, our study showed an improved and sustained response at the FU visit, whereas some previous studies evaluating various regimens of fluconazole have reported an 11–20% decrease in sustained response occurring from days 7–14 to days 28–35.^15^, ^17^, ^22^

Therapies for VVC have historically been limited to azoles, which are fungistatic. Ibrexafungerp has demonstrated preclinical activity in patients with azole‐ and echinocandin‐resistant *Candida* species.^23^, ^24^ Retention of this activity, even in fluconazole‐resistant isolates, at normal vaginal pH level (4.5) has been demonstrated and even enhanced in *Candida* isolates.^10^ In comparison, *in vitro* azole activity against *Candida* species has been shown to decrease with a drop in pH level from 7 to 4.5.^25^, ^26^ Furthermore, ibrexafungerp has demonstrated good vaginal penetration preclinically, with tissue levels 2‐ to 9‐fold higher than plasma levels, compared with ratios of 0.4–0.7 reported clinically with fluconazole.^10^, ^27^, ^28^ The retention of activity at lower pH levels, coupled with increased vaginal tissue penetration of ibrexafungerp and its fungicidal mechanism of action, may allow for improved treatment of VVC.

A higher percentage of patients receiving ibrexafungerp (14.8%) experienced a treatment‐related TEAE compared with those receiving placebo (4.0%). Ibrexafungerp was well tolerated, with most treatment‐related TEAEs being gastrointestinal disorders that were mild to moderate in nature. TEAEs that led to study discontinuation were not related to ibrexafungerp. Unlike other systemic azole therapies, hepatotoxicity and cardiac arrhythmias have not been reported with ibrexafungerp.^28^, ^29^, ^30^

## Conclusion

Ibrexafungerp demonstrated reproducible statistical superiority versus placebo in VVC treatment and results of this study support findings from the similarly designed VANISH 303 study. Future research of ibrexafungerp is warranted and should evaluate its effectiveness in cases of acute VVC with non‐*albicans Candida* species. In June 2021, ibrexafungerp (BREXAFEMME^®^) was approved as the first and only non‐azole treatment for VVC, thereby providing a new, oral, 1‐day treatment option for acute VVC that is well tolerated and effective. Ibrexafungerp is being evaluated in the treatment of recurrent VVC in a phase 3 study (CANDLE, NCT04029116).

### Disclosure of interests

RS reports personal fees (consultant/advisory board) from SCYNEXIS, Inc., and clinical study funds (received by institution) during the conduct of the study, and personal fees from Mycovia Pharmaceuticals, Inc., outside of the submitted work. PN reports personal fees (consultant) during the conduct of the study from SCYNEXIS, Inc., and personal fees (consultant) from Hologic, Inc. and Mycovia Pharmaceuticals, Inc. outside of the submitted work. MAG reports clincial study funds (received by institution) during conduct of the study and personal fees from SCYNEXIS, Inc. outside of the submitted work. NEA, DA and IAH are employed by and hold stocks in SCYNEXIS, Inc. KBE is a consultant with SCYNEXIS, Inc. DAD and JDS have no conflicts to disclose.

### Contribution to authorship

RS was involved as a principal investigator, assisted in analysis and interpretation of the data and formulating conclusions of the study, participated in writing of the manuscript, and provided final approval of the manuscript. PN was involved in reviewing and developing the study protocol development, reviewing the analyses of study data, reviewing and editing the manuscript, and final approval of the manuscript. MAG was involved in fungal isolation and identification of samples collected from patients as well as manuscript planning, review and final approval. DAD was involved as a principal investigator and provided reviews of manuscript drafts and final approval of the manuscript. NEA contributed to the study protocol development, the study conduct and execution, data analyses, manuscript development, and final approval of the manuscript. DA was responsible for the conception and oversight of the study design, implementation, and data analyses, and contributed to the writing of the clinical study report and manuscript and provided final approval of the manuscript. IAH was involved in the conception, planning and implementation of the study as well as analysing the data, writing of the manuscript, and final approval of the manuscript. KBE was responsible for the review and summary of the mycology data from the clinical study including review of laboratory procedures and study outputs, summary of the mycology data in the clinical study report, preparation of the mycology report that was filed in support of the study, and reviewing the manuscript and providing final approval of the manuscript. JDS was involved in study protocol development and implementation (served as a principal investigator) as well as manuscript writing, review and final approval.

### Details of ethics approval

The procedures of this study received ethics approval from the Republic of Bulgaria, Bulgarian Drug Agency (EKKI/CT‐0446), on 5 June 2019, and ADVARRA (Pro00033647) on 24 April 2019.

### Funding

The VANISH 306 study was funded by SCYNEXIS, Inc.

### Acknowledgements

We thank the patients, the investigators and the investigational staff of the VANISH 306 study. The authors acknowledge the medical writing assistance of Laura Jung, PharmD, of PRECISIONscientia in Yardley, Pennsylvania, which was supported financially by SCYNEXIS, Inc. in compliance with international Good Publication Practice guidelines.

## Supporting information

Supplementary Material

Supplementary Material

Supplementary Material

Supplementary Material

Supplementary Material

Supplementary Material

Supplementary Material

Supplementary Material

Supplementary Material

## Data Availability

Qualified scientific and medical researchers may make requests for individual participant data that underlie the results (text, tables, figures and supplements) reported in this article, after de‐identification, at medicalaffairs@SCYNEXIS.com. Methodologically sound proposals for such data will be evaluated and approved by SCYNEXIS, Inc. at its sole discretion. All approved researchers must sign a data access agreement before accessing the data. Data will be available as soon as possible but no later than within 1 year of the acceptance of the article for publication, and for 3 years following article publication. SCYNEXIS, Inc. will not share identified participant data or a data dictionary.
